# Population Genetic Analysis of the *Theileria annulata* Parasites Identified Limited Diversity and Multiplicity of Infection in the Vaccine From India

**DOI:** 10.3389/fmicb.2020.579929

**Published:** 2021-01-20

**Authors:** Sonti Roy, Vasundhra Bhandari, Madhumanti Barman, Pankaj Kumar, Vandna Bhanot, Jaspreet Singh Arora, Satparkash Singh, Paresh Sharma

**Affiliations:** ^1^National Institute of Animal Biotechnology, Hyderabad, India; ^2^Manipal Academy of Higher Education, Manipal, India; ^3^Division of Livestock and Fisheries Management, ICAR-Research Complex for Eastern Region, Patna, India; ^4^Disease Investigation Laboratory, Lala Lajpat Rai University of Veterinary and Animal Sciences, Ambala, India; ^5^School of Animal Biotechnology, Guru Angad Dev Veterinary and Animal Sciences University, Punjab, India

**Keywords:** genotyping, schizont stage vaccine, Theileria annulata, population genetics, genetic diversity

## Abstract

**Background:** Apicomplexan parasite *Theileria annulata* causes significant economic loss to the livestock industry in India and other tropical countries. In India, parasite control is mainly dependent on the live attenuated schizont vaccine and the drug buparvaquone. For effective disease control, it is essential to study the population structure and genetic diversity of the *Theileria annulata* field isolates and vaccine currently used in India.

**Methodology/Results:** A total of 125 *T. annulata* isolates were genotyped using 10 microsatellite markers from four states belonging to different geographical locations of India. Limited genetic diversity was observed in the vaccine isolates when compared to the parasites in the field; a level of geographical substructuring was evident in India. The number of genotypes observed per infection was highest in India when compared to other endemic countries, suggesting high transmission intensity and abundance of ticks in the country. A reduced panel of four markers can be used for future studies in these for surveillance of the *T. annulata* parasites in India.

**Conclusion:** High genetic variation between the parasite populations in the country suggests their successful spread in the field and could hamper the disease control programs. Our findings provide the baseline data for the diversity and population structure of *T. annulata* parasites from India. The low diversity in the vaccine advocates improving the current vaccine, possibly by increasing its heterozygosity. The reduced panel of the markers identified in this study will be helpful in monitoring parasite and its reintroduction after *Theileria* eradication.

## Introduction

Bovine theileriosis, a tick-borne infectious disease, remains a severe problem for livestock in tropical countries affecting millions of animals, especially crossbreed and exotic cattle annually. The dominant *Theileria* species, linked to economic loss and mortality worldwide, are *Theileria annulata* and *Theileria parva*. *Theileria annulata* is responsible for the majority of theileriosis cases in India, while there are no reports of *T. parva* from the country (George et al., 2015). The management of the disease is mainly dependent on a live attenuated *T. annulata* schizont vaccine and a hydroxynaphthoquinones class of drug Buparvaquone. High prevalence rates (3–41%) of *T.annulata* were reported from different states of India (Kundave et al., 2015; Kumar et al., 2016; Dandasena et al., 2018). The attenuated vaccine is 100% effective against the homologous parasite challenge; however, the efficiency decreases in the presence of heterologous parasites in the field (Gill et al., 1980; Hashemi-Fesharki, 1988; Darghouth et al., 1996). There is limited information on the genetic diversity and population structure of the *T. annulata* parasites prevalent in field and in vaccine from India. The present study was designed to study the population genetics of the *T. annulata* parasites in the country.

*Hyalonmma anatolicum* transmits *T. annulata* sporozoites in the host and causes a lymphoproliferative disease similar to cancer (Ghosh and Azhahianambi, 2007; Tretina et al., 2015). The *T. annulata* sporozoites transform to the schizont stage and reside inside the host lymphocyte and macrophage cells (Sager et al., 1998). Among the different parasite stages in the host, the schizont stage is the symptomatic stage, based on which attenuated schizont vaccines were designed. The cell culture-based attenuated vaccines have been used in countries, like India (Gupta et al., 1998), China (Zhang, 1997), Russia (Stepanova and Zablotskii, 1989), Turkey (Sayin et al., 1997), Spain (Viseras et al., 1997), Israel (Pipano and Tsur, 1966), Iran (Hashemi-Fesharki, 1988), Tunisia (Darghouth, 2008), and Morocco (Ouhelli et al., 1997) for controlling the *T. annulata* infection. The attenuation of the vaccine line because of the long term passage results in loss of genotypes, decreasing the diversity of the parasites (Darghouth et al., 1996; Pipano and Shkap, 2006; Bilgic et al., 2019). Previous studies in *T. parva* have also shown limited genetic and antigenic diversity in the Muguga cocktail vaccine, recommending modification in the current vaccine by enhancing its diversity (Hemmink et al., 2016). Genetic diversity studies have been shown to be important for dissecting important information about protozoan parasites, like epidemiology, control, evolution, virulence, antigenicity, infectivity, drug sensitivity, and host preference (Majewska and Sulima, 1999; Sivakumar et al., 2014).

The multilocus genotyping technique has been used for studying the genetic diversity, transmission dynamics, and population structure of *T. annulata* parasites from other endemic countries, like China, Oman, Turkey, Tunisia, Portugal, and Sudan (Weir et al., 2007, 2011; Al-Hamidhi et al., 2015; Gomes et al., 2016; Yin et al., 2018). High genetic diversity, presence of multiple genotypes per sample, and geographical sub-structuring were the highlighting feature of the *T. annulata* populations reported till now (Weir et al., 2007, 2011; Al-Hamidhi et al., 2015; Gomes et al., 2016; Yin et al., 2018). Recently using a small number of samples, our group has shown high allelic and antigenic diversity in the clinical strains of *T. annulata* from India (Roy et al., 2019). However, comparative population genetic analysis among the *T. annulata* vaccine and the field isolates from different geographical locations of India is not known.

In the present study, microsatellite-based genotyping has been used for understanding the genetic diversity, population structure, and geographical substructuring of the *T. annulata* vaccine and parasite isolates collected from the four different locations in India. The genotyping results were also compared with similar data from other endemic countries. The results provide the first insight into the population genetics and diversity of the *T. annulata* parasites in India.

## Materials and Methods

### Parasite Sample Collection

Blood samples were collected from the suspected animals from four different geographical locations of India (Telangana, Gujarat, Haryana, and Bihar). Approximately 3 ml of blood was collected in EDTA coated vacutainer tubes (BD) with the help of trained veterinarians. A total of 125 blood samples, including the *T. annulata* vaccine, were collected. DNA was isolated from the blood using the standard phenol chloroform isoamyl alcohol method. DNA concentration and integrity were checked using nanodrop, and by running 0.8% agarose gel in TAE buffer. Diagnosis of the *T. annulata* infection was done based on microscopic analysis of Giemsa stained smears and PCR using *T. annulata* specific primers. The primers used were specific to the *T. annulata* Surface protein (*TaSP*) gene of the parasite (Roy et al., 2019).

### Microsatellite Genotyping

Genotyping was done using the 10 microsatellite markers previously described for *T. annulata* (Weir et al., 2007). The forward primer used for amplifying the markers was labeled with FAM at the 5' end for detection in capillary electrophoresis. The DNA samples (*N* = 125) were used as a template for amplifying the 10 markers from each sample using a previously described protocol (Roy et al., 2019). Amplified PCR products were purified using the Qiagen PCR clean-up kit and stored in an amber tube to reduce the fluorescence loss. The amplified products were separated on the ABI 3730Xl electrophoresis instrument, with Liz500 as the standard internal marker for the fragment size analysis.

### Data Analysis

The file generated from capillary electrophoresis was imported into Gene marker 2.7.0 software (SoftGenetics, LLC) for further analysis. Allele scoring for each marker was done in the previously described range for *T. annulata*. The alleles were scored based on the predominant peak, and only peaks above 25% of this peak were recorded for analysis. Stutter peak filter was applied to remove stutter peaks within 2.5 base pairs of the primary peak. Plus A filter was used to prevent calling two alleles in case of a split peak, which is one base pair apart. The total number of alleles was counted and averaged for each sample across 10 loci to estimate the multiplicity of infection (MOI). Multilocus genotype (MLG) data for each sample were generated using the predominant peak (Weir et al., 2007). For comparative population study, publicly available similar data from other endemic countries were used for analysis (Weir et al., 2011; Al-Hamidhi et al., 2015). The MLG data were used to estimate the allele frequency, number of effective alleles (Ne), and expected heterozygosity (He) using GenAlEx 6.503 (Peakall and Smouse, 2012) as an excel add-in tool kit. The proportion of shared alleles was calculated using PopGenReport package in R software (Adamack and Gruber, 2014). Mean allele number and allele richness (*R_s_*) were calculated for all the markers using the FSTAT 2.9.4 program (Goudet, 2003). Lian 3.7 Program (Haubold and Hudson, 2000) was used to calculated I^A^_S_, V_D_, and V_para_ to predict linkage disequilibrium (LD) in the population using the MLG data. Population genetic differentiation (Fst) was calculated on the Genepop 4.2 Web Server (Raymond, 1995; Rousset, 2008) using the MLG data to understand the population differentiation. Bayesian analysis was done using the STRUCTURE 2.3.4 software (Pritchard et al., 2000; Evanno et al., 2005). Twenty iterations were run for each group (*K* = 1–12) with a burn-in of 50,000 steps and then 500,000 Bayesian Markov Chain Monte Carlo (MCMC) steps using the admixture model. The optimal number of clusters was identified using method described by Evanno et al. (2005). Discriminant Analysis of Principal Components (DAPC) was done by R software using the Adegenet 2.0.1 package (Jombart, 2008) for understanding the geographical substructuring between the populations using the MLG data.

The complete allelic profile of each sample was next used to prepare a binary data set for the presence and absence of alleles for understanding the genotypic diversity considering multiple parasite genotypes in each sample. A similarity matrix and dendrogram were created based on the allelic data by Jaccard’s similarity index using online server DendroUPGMA (Garcia-Vallve et al., 1999). Interactive Tree Of Life (iTOL) software was used for visualization of the tree (Letunic and Bork, 2007).

### Bottleneck Analysis

The Bottleneck analysis was done using the allele frequency data for assessing the change in the population size by measuring excess or deficit in heterozygosity by Bottleneck software version 1.2.02 (Piry et al., 1999). The two-phase model (TPM) with 1,000 iterations was used to compare the number of loci in population that present heterozygosity excess or deficiency under the mutation drift equilibrium (Cornuet and Luikart, 1996; Luikart and Cornuet, 1998). Sign test and Wilcoxon test were used for identifying the statistical significance of the data generated using TPM model.

Recent effective population size reductions (genetic bottlenecks) were studied using allele frequency data and BOTTLENECK software (Version 1.2.02; Cornuet and Luikart, 1996). To determine whether a population exhibits a significant number of loci with heterozygosity excess, BOTTLENECK proposes three tests: sign test, standardized differences test (minimum 20 loci), and Wilcoxon sign-rank test. Finally, the allele frequency distribution was established in order to see whether it is approximately L-shaped (as expected under mutation-drift equilibrium) or not (recent bottlenecks provoke a mode shift). As recommended by Piry et al. (1999), the TPM with 95% proportion of the Stepwise Mutation Model (SMM) and 5% of the Multistep mutations was used.

### Optimal Set of Marker for Population Differentiation

For identifying a minimum number of microsatellite markers for differentiating the parasite population, sequential removal of markers was done for counting haplotypes based on the He values starting from low to high. The optimal set of markers needed for population differentiation and the correlation between the populations of different states was done using Genalex 6.503 software (Peakall and Smouse, 2012; Kittichai et al., 2017).

### Ethics Statement

Collection of less than 5 ml of blood, in accordance with national legislation, is exempt from ethical approval requirements. The animal study was reviewed and approved by Institutional Animal Ethics committee, National Institute of Animal Biotechnology, Hyderabad.

## Results

### High Genetic Diversity and MOI in the *Theileria annulata* Isolates From India

**Figure 1:**
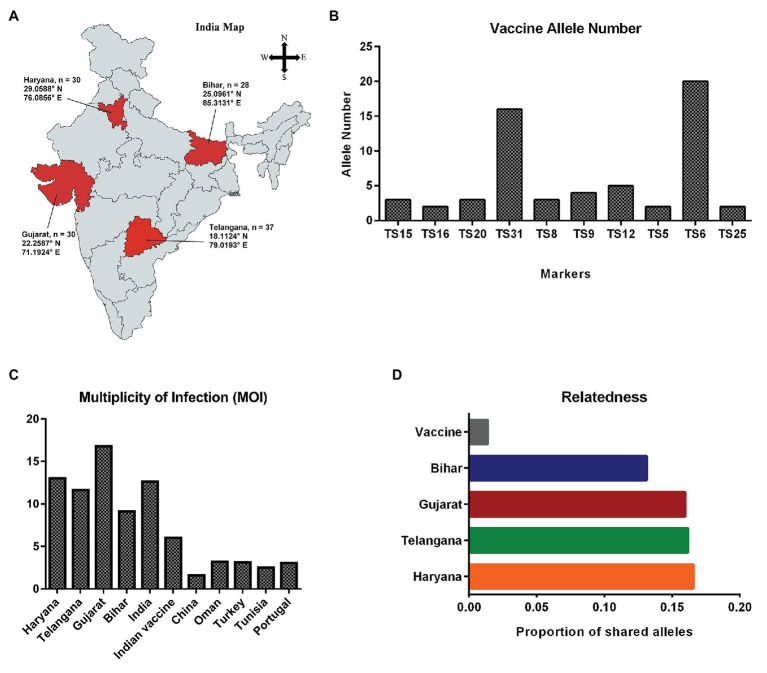
Sample collection map and genetic diversity: **(A)** Sampling locations of *Theileria annulata* are indicated on the map of India; **(B)** Graph showing total number of alleles detected for the 10 markers in vaccine; **(C)** Comparative analysis of multiplicity of infection (MOI) in *T. annulata* isolates from India and other endemic countries; and **(D)** Proportion of shared alleles between the *T. annulata* isolates of different states and vaccine.

**Figure 2:**
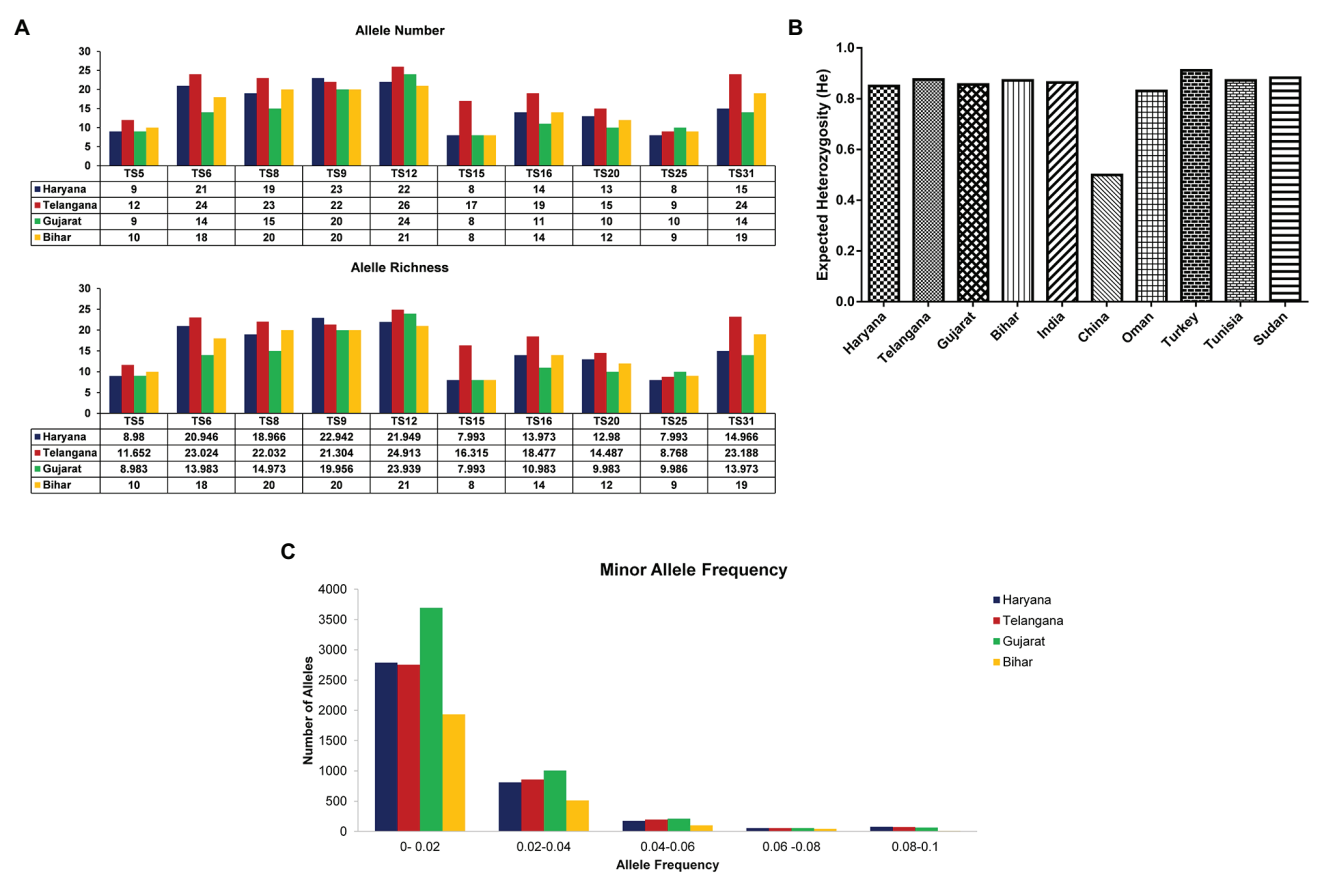
Allelic Number and Allele richness, Expected heterozygosity (He), and Minor allele frequency: **(A)** Graph showing number of alleles and allele richness of 10 markers in *T. annulata* isolates from four states in India; **(B)** Comparative analysis of expected heterozygosity (He) values between the *T. annulata* population of India and other endemic countries; and **(C)** The graph shows distribution of number of alleles against different frequency range in *T. annulata* isolates from four states in India.

A total of 125 samples were collected from four different states (Telangana, Haryana, Gujarat, and Bihar), including the vaccine for assessing genotypic diversity of *T. annulata* parasites in India (Figure 1A). The parasite infection was confirmed by Giemsa stained smears and PCR in all the samples. The genotypic diversity was studied using a panel of 10 micro and minisatellite markers in *T. annulata*. The diversity of the isolates was assessed based on their; allelic profile, MOI, allelic richness, the proportion of shared alleles, effective number of alleles, expected heterozygosity, and minor allele frequency. The allelic profile of the vaccine showed less than five alleles in most of the markers except TS6 and TS31 (Figure 1B). The genotyping showed the presence of mixed parasite infections in each isolate based on the presence of multiple alleles at each locus. The allelic data per locus were used for calculating MOI for each sample from all the states. The MOI values for Gujarat (16.77), Haryana (13.03), and Telangana (11.64) were found to be significantly higher when compared to Bihar (Table 1). The genotypes per infection in the vaccine line based on the MOI values (6) were substantially less than the parasite population from all the states (Figure 1C). The comparative MOI analysis between the different endemic countries showed very high number of genotypes per infection in Indian population (Figure 1C). Since multiple alleles were found in the samples, the MLG profile was created by selecting the predominant allele from each locus for all the samples. The MLG data identified 125 unique haplotypes with no sharing between or within the states. High polymorphism was found in all the markers in the field samples, with an overall number of alleles ranging from 26 (TS12) to 8 (TS25, TS15) per marker. Allele comparison between the vaccine and the regional isolates showed little proportion of alleles shared among them (Figure 1D). Out of 10 markers, TS8, TS9, TS12, and TS31 showed no shared allele with the vaccine, while TS5, TS6, TS15, TS16, TS20, and TS25 showed some shared alleles within the population. We next calculated mean allele number and allele richness using the MLG data (Table 1). Telangana showed the highest allele number (19.1) and allele richness (18.42) among the regional population, followed by Haryana, Bihar, and Gujarat. TS6, TS8, TS9, TS12, and TS31 markers showed more number of alleles per locus and also had high allele richness consistently in all the population (Figure 2A).

**Table 1:** Population Diversity.

Population	N	MOI^#^	Ne	Mean number of alleles	Mean allelic richness^a^	Mean H_E_
Haryana	30	13.03	10.465	15.2	15.17	0.850
Telangana	37	11.64	12.114	19.1	18.42	0.876
Gujarat	30	16.77	9.126	13.5	13.48	0.856
Bihar	28	9.15	10.895	15.1	15.10	0.872
Total	125	12.64	10.650	15.72	22.84^b^	0.864

a Allelic richness based on a minimum sample size of 28 haploid individual samples.

b Allelic richness based on a minimum sample size of 125 haploid individual samples.

# Significant by one-way ANOVA between Haryana and Gujarat, Haryana and Bihar, Telangana and Gujarat, Telangana and Bihar, and Gujarat and Bihar.

Expected Heterozygosity (H_E_) is high for all the population. Expected H_E_ measures will range from zero (no heterozygosity) to nearly 1.0.

The number of effective alleles ranged from 9.1 (Gujarat) to 12.1 (Telangana; Table 1). The MLG data were next used for calculating the expected heterozygosity between the states and in the total population. The expected He was found to be high (0.864) in samples across India (Table 1). We also compared expected He of parasite population from India to previously reported *T. annulata* isolates from countries, like Oman, Portugal, Sudan, Tunisia, China, and Turkey (Figure 2B). The high genetic diversity of Indian parasites was similar to other countries, like Oman, Turkey, Tunisia, and Sudan. Further allele frequency was calculated from the allelic data per locus for segregating the common and rare alleles, which in turn helps in identifying the diversity of the parasite population. The allele count was highest for Gujarat in the frequency range of 0–0.02 and 0.02–0.04, followed by Haryana, Telangana, and then Bihar suggesting high diversity in the former state (Figure 2C). As the frequency range increases, there is not much difference between the populations.

### Geographical Sub-structuring in the Indian *Theileria annulata* Parasite Population

**Figure 3:**
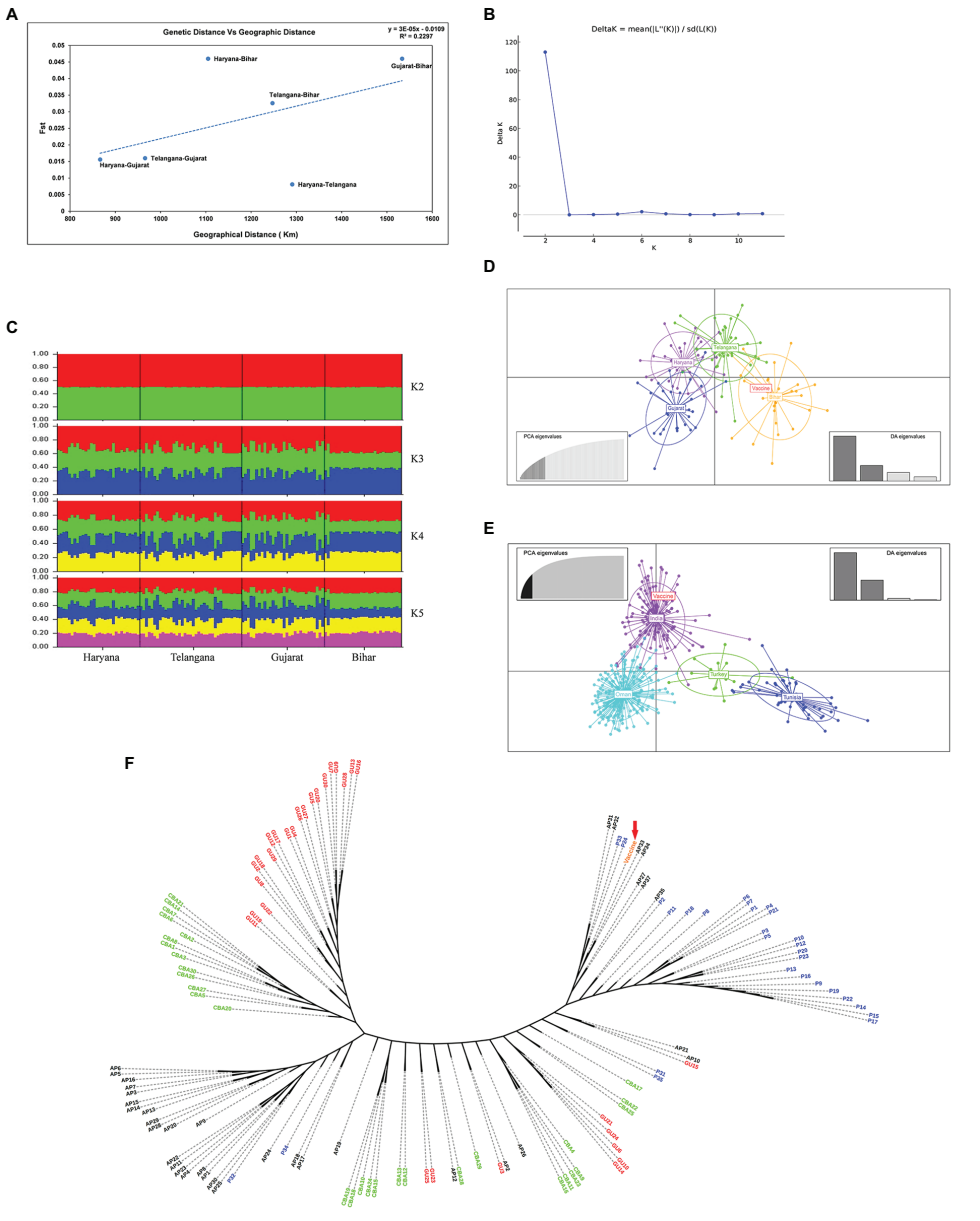
Population structure: **(A)** Graph showing correlation between pairwise genetic distance and geographic distance based on the Fst values among the four states; **(B)** The graph shows optimal number of clusters from the STRUCTURE analysis; **(C)** STRUCTURE analysis from *K* = 2 to *K* = 5 with isolates from the four states of India; **(D)** Discriminant Analysis of Principal Components (DAPC) analysis showing the genetic structure of *T. annulata* populations from four states; **(E)** DAPC analysis showing the genetic structure of *T. annulata* populations from India, Turkey, Tunisia, and Oman; and **(F)** A phylogenetic tree was drawn to show complete allelic profiling between the samples from Bihar (P1–P35), Haryana (CBA1–CBA30), Gujarat (GU1–GU30), and Telangana (AP1–AP30).

For understanding the genetic variance among the Indian *T. annulata* parasite population, pairwise Fst values were calculated using the MLG profiles. Based on the Fst data, genetic divergence was found higher in the Bihar parasite population when paired to Gujarat (0.046), Haryana (0.046), and Telangana (0.0326; Table 2). While low variance was observed between the parasite population of Haryana, Gujarat, and Telangana with Fst values ranging from 0.0081 to 0.0156. Next, we compared the Indian parasite population with the previously reported parasite population from countries like Oman, Turkey, Tunisia, and Sudan using their MLG profiles. The Fst data showed high genetic variance between the Indian and Tunisia (0.0850) parasite population. While moderate variation was seen when the pairwise comparison was made between the India and Sudan (0.0691), India and Oman (0.0647), and India and Turkey (0.0573) parasite populations. Next, we calculated Nei’s genetic distance between Indian populations for finding the relation between genetic and geographic distance. No significant correlation was observed when pairwise Nei genetic distance or Fst values were plotted against geographic distance in kilometer, with *R*^2^ value of 0.2889 and 0.2297, respectively (Figure 3A). Structure analysis using the Bayesian iterative algorithm did not show any clear pattern of distribution among the Indian parasite populations at *K* values ranging from *K* = 1 to *K* = 12 (Figures 3B,C). However, the DAPC analysis showed clustering between the parasite populations of different states, suggesting a level of geographical distribution in India (Figure 3D). The Indian vaccine clustered near to the Bihar and Telangana parasites in the analysis. The *T. annulata* parasite population was next compared using similar data to parasites from other endemic countries, like Tunisia, Turkey, and Oman. The DAPC analysis supported a high genetic differentiation between populations in India, Tunisia, Turkey, and Oman (Figure 3E). The differentiation analysis done until now is based on the predominant allele of the isolates and might miss other relevant alleles that might show differentiation between the populations. Therefore, we constructed a phylogenetic tree based on the complete allelic profiles of all the samples to understand the genetic differentiation pattern. The analysis showed regional clustering in the samples from Haryana, Telangana, and Gujarat, although some samples had mixed distribution within other regions (Figure 3F). The vaccine strain was found near to the isolates from state of Bihar and Telangana, suggesting some level of similarity among the parasites.

**Table 2:** Pairwise Fst values between all geographically isolated populations.

Comparison between	N	Fst
Haryana and Telangana	67	0.0081
Haryana and Gujarat	60	0.0156
Haryana and Bihar	58	0.046
Telangana and Gujarat	67	0.0160
Telangana and Bihar	65	0.0326
Gujrat and Bihar	58	0.046
India and Oman	356	0.0647
India and Turkey	138	0.0573
India and Tunisia	174	0.0850
India and Sudan	129	0.0691

N, Number of Individuals; Fst, Fixative Index (measure of Genetic Differentiation).

### LD and Nonbottlenecked Parasite Population in India

**Figure 4:**
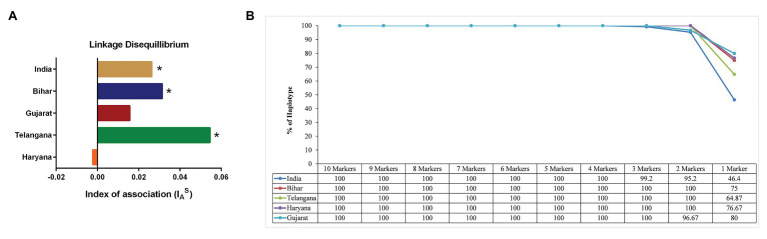
Linkage Disequilibrium (LD) and Minimum number of Markers for complete Haplotype Detection: **(A)** The graph shows the standardized index of association I_A_^S^ values of the *T. annulata* population from the states of Haryana, Telangana, Bihar, and Gujarat. Higher I_A_^S^ indicates non-random association between loci. (^*^p < 0.01). **(B)** The figure shows percentage of haplotypes detected in the *T. annulata* populations by sequential removal of markers based on the He values.

The MLG data was next used for identifying LD in the Indian parasite population by calculating the standard index of association (I^S^_A_). We found significant LD in parasite population of Telangana (I^S^_A_ = 0.0545, *p* < 0.001), Bihar (I^S^_A_ = 0.0313, *p* < 0.001) and in total Indian population (I^S^_A_ = 0.0263, *p* < 0.001; Supplementary Table S1). However, in the state of Haryana and Gujarat, I^S^_A_ values were found to be statistically insignificant to conclude anything [I^S^_A_ = −0.0022, *p* = 6.70 × 10^−01^, I^S^_A_ = 0.0156, *p* = 1.10 × 10^−01^, respectively (Figure 4A)]. We observed LD when comparing Indian parasite population in combination with other countries population, like Oman (I^S^_A_ = 0.0313, *p* < 0.001), Turkey (I^S^_A_ = 0.0313, *p* < 0.001), Tunisia (I^S^_A_ = 0.0313, *p* < 0.001), Sudan (I^S^_A_ = 0.0313, *p* = < 0.001), and India and other countries (I^S^_A_ = 0.0429, *p* < 0.001). Further bottleneck analysis was done using allele frequency data for checking the recent population size reduction in the Indian parasites under the TPM model. Statistical analysis of the data was carried out, under the assumption of mutation-drift equilibrium, by Sign rank test and the Wilcoxon test. A significant heterozygosity deficit was seen in the population, indicating nonbottlenecked parasite population in India (Supplementary Table S2).

### Minimum Four Microsatellite Markers Can Be Used for Differentiating the Parasite Population

The next question we asked was whether less than 10 markers could be used for differentiating the parasite populations in these states of India. The microsatellite markers were removed from each sample sequentially based on their He values from low to high, keeping the highly diverse markers for counting the number of haplotypes (Supplementary Table S3). We found that four markers (TS6, TS8, TS9, and TS12) were sufficient for detecting 100% haplotypes in the Indian population (Figure 4B). However, in a regional population of Gujarat (TS6, TS9, and TS12), three markers were enough, while Telangana (TS12, TS31), Haryana (TS9, TS12), and Bihar (TS9, TS12) two markers were able to detect all haplotypes.

## Discussion

This study is the first comprehensive analysis of the *T. annulata* diversity and population structure from India using microsatellite typing. The samples (*N* = 125) for the study were collected from four different geographical locations, including the *T. annulata* vaccine from India. The genotyping analysis identified high diversity among the microsatellite makers and the presence of multiple genotypes in all the samples. Based on the analysis, we found less genetic diversity in the *T. annulata* vaccine when compared to the parasite population from the field.

The total number of alleles per locus was found to be very low in the vaccine in comparison to the field isolates indicating limited diversity. High genetic diversity (H_E_; 0.864) was found in the parasite population of all four locations; similar diversity was reported from other endemic countries, like Turkey, Tunisia, Oman, and Sudan (Weir et al., 2007, 2011; Al-Hamidhi et al., 2015). However, diversity was very high when compared to the parasite population from China and Portugal (Gomes et al., 2016; Yin et al., 2018). Based on the previous reports where a direct relationship has been shown between genetic diversity and effective population size, it can be concluded that the effective population size of *T. annulata* is high in India (Al-Hamidhi et al., 2015). The MOI values (9.15–16.77) indicated the presence of mixed genotypes in each sample, including vaccine (MOI = 6). Among the four states, Bihar has the lowest, and Gujarat has the highest MOI, followed by Haryana and Telangana. Compared to the MOI values reported from the other endemic countries, the number of genotypes present in a single infection was highest in India (Weir et al., 2007, 2011; Al-Hamidhi et al., 2015; Gomes et al., 2016; Yin et al., 2018). The significantly different MOI values inside India and when compared to other countries might be linked to factors, such as an abundance of the tick vector, time of sample collection, and transmission intensity. The high MOI in India points toward high transmission intensity and vector abundance in the country (Al-Hamidhi et al., 2015). The allele frequency data based on the presence of less frequent alleles indicated that parasite population in Gujarat is highly diverse among the four states with Bihar having the lowest diversity (Manske et al., 2012). The MOI values of the clinical samples were found to be very high when compared to the Indian vaccine strain (MOI = 6), suggesting less parasite diversity in the vaccine. The presence of multiple parasites in the infected sample points toward random mating in the tick host; however, the level of cross mating and recombination could not be determined (Hill et al., 1995). We then estimated the inbreeding coefficient 0.08 (*f* = 1/ne) and extent of outcrossing (>50%) for the *T. annulata* population in India using the mean number of clones per infection (ne = 12.64). This method is prevalidated in similar kinds of studies on *T. annulata* and *Plasmodium falciparum* (Hill et al., 1995; Al-Hamidhi et al., 2015). High outcrossing events found in our study have been previously linked to the formation of new genotypes different from the vaccine types, leading to its reduced efficacy in the field.

Despite high genetic diversity in samples from all the four sites, significant LD was found in the *T. annulata* populations from the states of Telangana and Bihar. The significant LD was also observed when all the samples were considered as one population. Although LD was observed in India, its values were not in the range where population structure can be considered to be clonal. The high genetic diversity and LD have been previously reported for *T. annulata* from other endemic countries and other similar apicomplexan parasites, like *T. parva*, and *P. falciparum* (Muleya et al., 2012; Al-Hamidhi et al., 2015; Wei et al., 2015; Yin et al., 2018). Observed LD can be because of multiple reasons, such as genetic drift, gene flow, and population size change. To detect population size change, we performed a bottleneck analysis, which showed no recent size reduction in India.

The level of genetic differentiation was found to be low when the pairwise comparison was done between state-wise parasite populations in India (Fst < 0.05). Moderate to high genetic differentiation was observed when the Indian parasite population was compared with other countries, like Oman, Turkey, Tunisia, and Sudan. The parasite population from Bihar was found to be genetically distant based on the Fst analysis when compared to the other three states. The low genetic differentiation between the parasites may be connected to the free movement of the animals in the country for commercial purposes. No correlation was found between the parasites based on the geographic and genetic distance (Fst). There was some evidence of regional distribution in the parasite population based on the clusters formed in the DAPC analysis. The current vaccine should ideally comprise of sufficient genotypic diversity for protection in all the states; however, it clustered near to the Bihar and Telangana population, signifying the presence of heterologous parasites in the field. The heterologous parasites in the field might be due to the frequent movement of animals inside and between the states, which provides an opportunity for the development of diverse parasites, making the population highly complex in India. The DAPC analysis reconfirmed the high genetic differentiation of the parasite population from India and other countries (Oman, Tunisia, and Turkey), which is evident as the animal movements between the countries are zero to none. As multiple parasites were present in every infection, an analysis based on the predominant allele might be biased. The phylogenetic analysis based on the complete allelic profiles of each sample also identified regional clustering in India. This implies that the genotype circulating in a particular region is similar and did not exactly match with other areas. This also proves that even though there is an allelic similarity among different states based on MLG, the genotypes circulating in different states are different. It might have a significant implication in parasite control as different genotypes may respond differently to control measures, and in the future, state-wise control strategies should be adopted. Therefore, diversity and genotypes circulating in a particular region have to be considered while implementing control measures.

The previous studies from other countries, have utilized 10 microsatellite markers for understanding the *T. annulata* population diversity. However, in the future, if we plan to track the parasite control measures in these states, such studies might not be feasible due to time and economics involved. Based on our genotyping data, we next checked whether it is possible to use a smaller number of markers for future genetic diversity studies. We identified that a minimum of four markers could be used to study the population structure of the parasite without missing the critical information and reducing the cost of the assay.

## Conclusion

Our study helps to understand the population structure of *T. annulata* parasites in India. The low genetic diversity observed in the vaccine highlights the scope for improvement in the current vaccine. The detection of multiple unique genotypes other than the vaccine, calls for increased efforts for *Theileria* control. We hypothesize that using a cocktail of parasites having sufficient genetic and antigenic diversity might be a good idea for the future vaccine. For a better understanding of the genotypic and antigenic composition of the parasite populations in the country, sample numbers and sites will have to be increased substantially in future studies. Our results can be used as the baseline data for future studies and will be helpful in monitoring the parasite population in the country. Our findings for the use of a smaller number of markers for genotype allocation in these states will be useful for the *Theileria* control programs.

## Data Availability Statement

The original contributions presented in the study are included in the article/Supplementary Material, further inquiries can be directed to the corresponding author.

## Author Contributions

PS and SR designed the experiments. SR, MB, and VBd have done the experiments and data analysis. VBo, PK, JA, and SS have helped in sample collection, designing study, data analysis, and manuscript editing. PS, SR, and VBd edited the paper. All authors have given approval to the final version of the manuscript.

### Conflict of Interest

The authors declare that the research was conducted in the absence of any commercial or financial relationships that could be construed as a potential conflict of interest.
