# Supplementary material for: Population Genetic Analysis of the Theileria annulata Parasites Identified Limited Diversity and Multiplicity of Infection in the Vaccine From India
Source: Front Microbiol. 2021 Jan 20;11:579929. doi: 10.3389/fmicb.2020.579929 (PMC7854550; doi:10.3389/fmicb.2020.579929)
Supplement: Supplementary file 1 [file Data_Sheet_1.docx]

**Table S1: Linkage Equilibrium analysis**

| Comparison | N | I^A^_S_ | P-Value | V_D_ | Ve | L_MC_ | L_PARA_ | Linkage |
| --- | --- | --- | --- | --- | --- | --- | --- | --- |
| Haryana | 30 | -0.0022 | 5.62 x 10^-01^ | 0.911 | 0.9295 | 1.0861 | 1.0669 | LE |
| Telangana | 37 | 0.0545 | < 1.00 x 10^-03^ | 1.3007 | 0.8131 | 0.9308 | 0.9173 | LD |
| Gujarat | 30 | 0.0156 | 3.80 x 10^-02^ | 1.0948 | 0.9601 | 1.0856 | 1.0752 | LD |
| Bihar | 28 | 0.0313 | < 1.00 x 10^-03^ | 1.0009 | 0.7809 | 0.8895 | 0.8789 | LD |
| India | 125 | 0.0263 | < 1.00 x 10^-03^ | 0.9363 | 0.7571 | 0.8003 | 0.7963 | LD |
| India and Oman | 356 | 0.0363 | < 1.00 x 10^-03^ | 1.2052 | 0.9085 | 0.9335 | 0.9331 | LD |
| India and Turkey | 138 | 0.0292 | < 1.00 x 10^-03^ | 0.8740 | 0.6922 | 0.7227 | 0.7245 | LD |
| India and Tunisia | 174 | 0.0355 | < 1.00 x 10^-03^ | 0.7860 | 0.5956 | 0.6132 | 0.6129 | LD |
| India and Sudan | 129 | 0.0271 | < 1.00 x 10^-03^ | 0.9140 | 0.7347 | 0.7738 | 0.7716 | LD |
| India, Oman, Turkey, Tunisia & Sudan | 422 | 0.0429 | < 1.00 x 10^-03^ | 1.0686 | 0.7708 | 0.7871 | 0.7879 | LD |

N: Number of Individuals, I^S^_A_: Standardized index of association, V_D_: Observed mismatch variance, Ve : Expected mismatch variance, L_MC_ - upper 95 % confidence limits of Monte Carlo simulation, L_PARA_ : parametric tests, LD : Linkage Disequilibrium, LE : Linkage Equilibrium

**Table S2: Bottleneck analysis.**

| SIGN Test | TPM | *P* value |
| --- | --- | --- |
| Expected number of loci with heterozygosity excess: | 6.39 | NA |
| Loci heterozygosity excess | 2 | NA |
| loci with heterozygosity deficiency | 8 | NA |
| Wilcoxon Test |  |  |
| Probability (one tail for H deficiency) | 0.00488 | < 0.01 |
| Probability (one tail for H excess) | 0.99658 | NS |
| Probability (two tails for H excess or deficiency) | 0.00977 | < 0.01 |

TPM – Two phase model, NS – Not significant, NA – Not applicable

**Table S3** - Haplotypes (% and number) detected after stepwise removal of markers for combined populations and individual populations from Bihar, Telangana, Gujarat and Haryana.

| **India** | | | | | |
| --- | --- | --- | --- | --- | --- |
| **Marker Number** | **Marker Removed** | **n** | **Haplotype** | **Haplotype %** | **Panel** |
| 10 | All Present | 125 | 125 | 100 | TS5, TS6, TS8, TS9, TS12, TS15, TS16, TS20, TS25, TS31 |
| 9 | TS25 | 125 | 125 | 100 | TS5, TS6, TS8, TS9, TS12 TS15, TS16, TS20, TS31 |
| 8 | TS5 | 125 | 125 | 100 | TS6, TS8, TS9, TS12, TS15, TS16, TS20, TS31 |
| 7 | TS15 | 125 | 125 | 100 | TS6, TS8, TS9, TS12, TS16, TS20, TS31 |
| 6 | TS20 | 125 | 125 | 100 | TS6, TS8, TS9, TS12, TS16, TS31 |
| 5 | TS16 | 125 | 125 | 100 | TS6, TS8, TS9, TS12, TS31 |
| 4 | TS31 | 125 | 125 | 100 | TS6, TS8, TS9, TS12 |
| 3 | TS6 | 125 | 124 | 99.2 | TS8, TS9, TS12 |
| 2 | TS8 | 125 | 119 | 95.2 | TS9, TS12 |
| 1 | TS9 | 125 | 58 | 46.4 | TS12 |

| **Bihar** | | | | | |
| --- | --- | --- | --- | --- | --- |
| **Marker Number** | **Marker Removed** | **n** | **Haplotype** | **Haplotype %** | **Panel** |
| 10 | All Present | 28 | 28 | 100 | TS5, TS6, TS8, TS9, TS12, TS15, TS16, TS20, TS25, TS31 |
| 9 | TS25 | 28 | 28 | 100 | TS5, TS6, TS8, TS9, TS12, TS15, TS16, TS20, TS31 |
| 8 | TS5 | 28 | 28 | 100 | TS6, TS8, TS9, TS12, TS15, TS16, TS20, TS31 |
| 7 | TS15 | 28 | 28 | 100 | TS6, TS8, TS9, TS12, TS16, TS20, TS31 |
| 6 | TS20 | 28 | 28 | 100 | TS6, TS8, TS9, TS12, TS16, TS31 |
| 5 | TS16 | 28 | 28 | 100 | TS6, TS8, TS9, TS12, TS31 |
| 4 | TS6 | 28 | 28 | 100 | TS8, TS9, TS12, TS31 |
| 3 | TS8 | 28 | 28 | 100 | TS9, TS12, TS31 |
| 2 | TS31 | 28 | 28 | 100 | TS9, TS12 |
| 1 | TS9 | 28 | 21 | 75 | TS12 |

| **Telangana** | | | | | |
| --- | --- | --- | --- | --- | --- |
| **Marker Number** | **Marker Removed** | **n** | **Haplotype** | **Haplotype %** | **Panel** |
| 10 | All Present | 37 | 37 | 100 | TS5, TS6, TS8, TS9, TS12, TS15, TS16, TS20, TS25, TS31 |
| 9 | TS25 | 37 | 37 | 100 | TS5, TS6, TS8, TS9, TS12, TS15, TS16, TS20, TS31 |
| 8 | TS5 | 37 | 37 | 100 | TS6, TS8, TS9, TS12, TS15, TS16, TS20, TS31 |
| 7 | TS15 | 37 | 37 | 100 | TS6, TS8, TS9, TS12, TS16, TS20, TS31 |
| 6 | TS20 | 37 | 37 | 100 | TS6, TS8, TS9, TS12, TS16, TS31 |
| 5 | TS16 | 37 | 37 | 100 | TS6, TS8, TS9, TS12, TS31 |
| 4 | TS6 | 37 | 37 | 100 | TS8, TS9, TS12, TS31 |
| 3 | TS8 | 37 | 37 | 100 | TS9, TS12, TS31 |
| 2 | TS9 | 37 | 37 | 100 | TS12, TS31 |
| 1 | TS12 | 37 | 24 | 64.86 | TS31 |

| **Haryana** | | | | | |
| --- | --- | --- | --- | --- | --- |
| **Marker Number** | **Marker Removed** | **n** | **Haplotype** | **Haplotype %** | **Panel** |
| 10 | All Present | 30 | 30 | 100 | TS5, TS6, TS8, TS9, TS12, TS15, TS16, TS20, TS25, TS31 |
| 9 | TS5 | 30 | 30 | 100 | TS6, TS8, TS9, TS12, TS15, TS16, TS20, TS25, TS31 |
| 8 | TS25 | 30 | 30 | 100 | TS6, TS8, TS9, TS12, TS15, TS16, TS20, TS31 |
| 7 | TS15 | 30 | 30 | 100 | TS6, TS8, TS9, TS12, TS16, TS20, TS31 |
| 6 | TS16 | 30 | 30 | 100 | TS6, TS8, TS9, TS12, TS20, TS31 |
| 5 | TS20 | 30 | 30 | 100 | TS6, TS8, TS9, TS12, TS31 |
| 4 | TS31 | 30 | 30 | 100 | TS6, TS8, TS9, TS12 |
| 3 | TS6 | 30 | 30 | 100 | TS8, TS9, TS12 |
| 2 | TS8 | 30 | 30 | 100 | TS9, TS12 |
| 1 | TS12 | 30 | 23 | 76.66666667 | TS9 |

| **Gujarat** | | | | | |
| --- | --- | --- | --- | --- | --- |
| **Marker Number** | **Marker Removed** | **n** | **Haplotype** | **Haplotype %** | **Panel** |
| 10 | All Present | 30 | 30 | 100 | TS5, TS6, TS8, TS9, TS12, TS15, TS16, TS20, TS25, TS31 |
| 9 | TS15 | 30 | 30 | 100 | TS5, TS6, TS8, TS9, TS12, TS16, TS20, TS25, TS31 |
| 8 | TS5 | 30 | 30 | 100 | TS6, TS8, TS9, TS12, TS16, TS20, TS25, TS31 |
| 7 | TS25 | 30 | 30 | 100 | TS6, TS8, TS9, TS12, TS16, TS20, TS31 |
| 6 | TS20 | 30 | 30 | 100 | TS6, TS8, TS9, TS12, TS16, TS31 |
| 5 | TS16 | 30 | 30 | 100 | TS6, TS8, TS9, TS12, TS31 |
| 4 | TS31 | 30 | 30 | 100 | TS6, TS8, TS9, TS12 |
| 3 | TS8 | 30 | 30 | 100 | TS6, TS9, TS12 |
| 2 | TS6 | 30 | 29 | 96.66666667 | TS9, TS12 |
| 1 | TS9 | 30 | 24 | 80 | TS12 |
